# Methylated circulating tumor DNA as a biomarker for colorectal cancer diagnosis, prognosis, and prediction

**DOI:** 10.1186/s13148-021-01095-5

**Published:** 2021-05-17

**Authors:** Farah J. Nassar, Zahraa S. Msheik, Rihab R. Nasr, Sally N. Temraz

**Affiliations:** 1 Department of Internal Medicine, Faculty of Medicine, American University of Beirut, P.O. Box: 11-0236, Beirut, Lebanon; 2 Department of Anatomy, Cell Biology and Physiological Sciences, Faculty of Medicine, American University of Beirut, P.O. Box: 11-0236, Beirut, Lebanon

**Keywords:** Methylation, Colorectal cancer, Circulating biomarker, Diagnosis, Prognosis and prediction

## Abstract

Worldwide, colorectal cancer (CRC) is a deadly disease whose death rate ranks second among cancers though its incidence ranks third. Early CRC detection is key and is associated with improved survival outcomes. However, existing tests for CRC diagnosis have several weaknesses thus rendering them inefficient. Moreover, reliable prognostic tests that can predict the overall cancer outcome and recurrence of the disease as well as predictive markers that can assess effectiveness of therapy are still lacking. Thus, shifting to noninvasive liquid biopsy or blood-based biomarkers is vital to improving CRC diagnosis, prognosis, and prediction. Methylated circulating tumor DNA (ctDNA) has gained increased attention as a type of liquid biopsy that is tumor-derived fragmented DNA with epigenetic alterations. Methylated ctDNA are more consistently present in blood of cancer patients as compared to mutated ctDNA. Hence, methylated ctDNA serves as a potential biomarker for CRC that is worth investigating. In this review, we explore what has been reported about methylated ctDNA as a biomarker for CRC diagnosis that can distinguish between CRC patients or those having adenoma and healthy controls as validated specifically through ROC curves. We also examine methylated ctDNA as a biomarker for CRC prognosis and prediction as confirmed through robust statistical analyses. Finally, we discuss the major technical challenges that limits the use of methylated ctDNA for clinical application and suggest possible recommendations to enhance its usage.

## Introduction

Colorectal cancer (CRC) is considered the third most commonly diagnosed (10.2%) and the second most fatal (9.2%) cancer worldwide among both sexes combined [1]. In 2018, an estimated 1.8 million new cases and 881,000 deaths were attributed to CRC. Even though the death rate is relatively high from this cancer, detection at an early stage is associated with better survival outcomes. Based on the Surveillance, Epidemiology, and End Results (SEER) Program of the National Cancer Institute (NCI) between 2010 and 2016, the percentage of diagnosed cases by stage was 38% for localized stage, 33% for regional stage, and 22% for distant stage and their reported 5-year relative survival by stage was 90.2%, 71.8%, and 14.3%, respectively. Screening helps the diagnosis of asymptomatic CRC which are less advanced than the symptomatic ones. Present diagnostic, prognostic, and therapy predictive tests for CRC have drawbacks that affect their success. This highlights the need of novel more effective noninvasive biomarkers for CRC early detection before it progresses to distant stage as well as biomarkers for its prognosis for surveillance of recurrence or progression during treatment.

Current research in oncology is directed at finding and evaluating biomarkers defined as biological characteristics that act as an indicator of normal biological process, carcinogenesis, or pharmacological response to a therapeutic intervention. Since solid biopsy is an invasive method that is dynamically affected by tumor heterogeneity and since radiology screening methods can pose a threat of exposure to excessive ionizing radiation, liquid biopsy as a noninvasive technique for sampling and analyzing of blood has been favored for biomarker detection. Biomarkers that are currently in use or under investigation in liquid biopsies include proteins, circulating tumor cells (CTC), circulating tumor DNA (ctDNA), circulating cell-free RNA, and exosomes [2–4]. ctDNA are of a particular interest in CRC since these fragmented DNA are readily available and offer a minimally invasive approach for tumor detection and characterization in circulation. It is postulated that ctDNA may have arisen from tumor microenvironment cells or from neoplastic tumor cells through necrosis, apoptosis, phagocytosis, or active release in the form of exosomes or lipoproteonucleotidic complex [5, 6]. These ctDNA can be distinguished from circulating DNA originating from healthy cells by the presence of genomic aberrations that correspond to those found in the tumor, such as tumor-specific mutations or methylation. However, somatic mutations could be compromised since CRC is a heterogeneous disease and the mutations might occur at low frequency [7, 8]. This could be overcome by detecting the epigenetic alterations of ctDNA such as DNA methylation which involves the addition of a methyl group to position 5 of the DNA cytosine ring by DNA methyltransferase enzymes and usually causes gene silencing. Interestingly, aberrant ctDNA methylation can be detected in circulation in different cancers and these ctDNA covalent modifications are more frequent and usually goes ahead many mutational changes at early stage of carcinogenesis [9]. As such, all of these characteristics along with the ease in detection render methylated ctDNA competent to be investigated as circulating biomarker. In this review, we are highlighting the studies that investigate the performance of circulating ctDNA methylation as a potential epigenetic biomarker for CRC diagnosis, prognosis, and prediction using robust statistical analysis to be translated for clinical application.

## Methodology

A literature search was performed on PubMed using the following search query for each section:

- Section A: colorectal cancer AND methylation AND diagnosis AND circulating
- Section B: colorectal cancer AND methylation AND prognosis AND circulating, colorectal cancer AND methylation AND prediction AND circulating

Most of the studies discussing circulating methylated ctDNA as biomarkers for CRC diagnosis report only sensitivity and specificity that is bound by the selected cutoff point that have been considered. Accordingly, in our search in section A, we wanted to highlight the studies that can assess the performance of methylated ctDNA as biomarkers under different cutoff points using receiver operating characteristic (ROC) curve. As for reporting circulating methylated ctDNA as CRC prognostic biomarkers in section B, we focused on studies that reported prognostic and predictive ctDNA through robust statistical analysis according to Reporting Recommendations for Tumor Marker Prognostic Studies (REMARK) checklist [10]. This checklist addressed widespread deficiencies in the reporting of prognostic biomarker studies and particularly included details about statistical analysis showing the relation between the biomarker and the outcome (such as univariate and multivariate analysis with estimated effect like hazard ratio and survival probability).

### DNA methylation as a biomarker for CRC diagnosis

Multiple screening tests for the detection of CRC have shown promising results in decreasing CRC incidence and mortality. Two important characteristics of a test are the sensitivity (or true-positive rate) and specificity (or true-negative rate) [11]. Currently, the gold-standard tool for CRC screening is colonoscopy. Although it is characterized by its high sensitivity in detecting CRC (> 95%) and precancerous lesions (88–98% for advanced adenoma (AA)), it has some limitations including the invasiveness of the method, unpleasant lengthy bowel preparation, discomfort during the test, probability of gut perforation, infection transmission, sedation and high cost, all of which affect the patient’s compliance [11, 12]. Sigmoidoscopy and computed tomography colonography (CT colonography) are other semi-invasive screening tests that show high sensitivity for detecting CRC (> 95% and > 90%, respectively) and precancerous lesions, but are also limited by unpleasant bowel preparation [11, 12]. Noninvasive inexpensive methods include fecal immunohistochemical test (FIT) that shows higher sensitivity in detecting CRC (60–85%) than fecal occult blood test (FOBT) that shows low sensitivity in detecting CRC (33–75%) due to dietary restrictions and the need for multiple sampling. Although both show poor detection of precancerous lesions and high rates of false positives and false negatives, patients are easy to accept these methods because of their noninvasive characteristics and low cost. In addition, several tumor markers such as carcinoembryonic antigen (CEA) and carbohydrate antigen (CA19-9) have been utilized in clinical practice but show unsatisfactory results for CRC diagnosis [12]. Novel, noninvasive, sensitive tests, such as those based on the detection of aberrant DNA methylation markers in the plasma or serum have emerged to detect CRC and precancerous lesions (Table 1).

**Table 1:** Diagnostic methylated ctDNA in colorectal cancer patients

Gene	Country	No. of cases	No. of controls	Cases characteristics	Sample type	Technology used	AUC (*p*-value)	Sensitivity (%)	Specificity (%)	References
*SEPT9*	China	117 CRC, 45 AA, 50 NAA	70 N	I (20), II (47), III (35), IV (4)	Plasma	Epi proColon 2.0		CRC (73.2), A (< 27.6), NAA (26.5), I (52.6), II (84.8), III (78.8), IV (100)	CRC (66.7)	[18]
*SEPT9*	China	63 CRC, 82 A&P	11 nonCRC GID, 494 NED		Plasma	Epi proColon 2.0	0.835	CRC (73), A&P (17.1), nonCRC GID (18.2)	CRC (94.5), A&P (94.5)	[19]
*SEPT9*	Singapore	26 CRC	26 N	All stage I	Serum	MethyLight	0.793 (< 0.001)	50	at least 90	[20]
*EYA4*							0.789 (< 0.001)	57.7	at least 90	
*TAC1*							0.768 (< 0.001)	57.7	at least 90	
*TAC1 and EYA4*							0.821 (< 0.001)	84.6	80.8	
*TAC1 and SEPT9*							0.851 (< 0.001)	73.1	92.3	
*SEPT9*	China	187 CRC, 25 P	109 N		Plasma	MethyLight	CRC (0.777)	CRC (62.6), I–II (57.1), III–IV (69.5), P (12)	91.7	[21]
*OSMR*							CRC (0.796)	CRC (74.9), I–II (74.4), III–IV (76.8), P (20)	86.2	
*SEPT9 and OSMR*								CRC (77), I–II (78.1), III–IV (76.8), P (28)	81.7	
*TMEFF2*	Germany	133 CRC	179 N	I (20), II (32), III (47), IV (31)	Plasma	HM	0.72	all (30), I (5), II (22), III (34), IV (45)	95	[22]
*NGFR*							0.7	all (33), I (20), II (25), III (36), IV (36)	95	
*SEPT9*							0.8	all (52), I (30), II (56), III (45), IV (68)	95	
*SEPT9 and ALX4*	Germany	VC: 5 CRC, 49 P	VC: 22 N	VC: UICC I (4), III (1)	Plasma	MethyLight		P (71)	P (95)	[23]
*SEPT9*	China	98 CRC, 101 A	253 NED	0 (3), I (23), II (31), III (8), IV (2)	Plasma	Epi proColon 2.0	CRC: 0.802, A: 0.532 (< 0.001)	CRC (61.22), A (7.9)	CRC (98.42)	[24]
		98 CRC	253 NED, 101 A, 76 nonCRC cancers, 30 INF					61.22	93.7	
*SEPT9*	China	123 CRC	125 N	I (5), II (36), III (58), IV (4)	Plasma	MSP	all: 0.757	all (61.8), I (60), II (52.8), III (63.8), IV (50)	89.6	[25]
*SEPT9*	Taiwan	51 CRC	9 N	0-II (23), III–IV (28)	Plasma	Abbott MS-9		47	89	[26]
*SEPT9*	China	85 CRC, 364 A, 216 HPP, 372 GID	324 N	I (9), II (38), III (31), IV (7), SA (36), TA (202), TVA (108), VA (18)	Plasma	Epi proColon 2.0 CE	CRC: 0.887, AA: 0.675, NAA: 0.582, P: 0.555	all (87.1), I (77.8), II (78.9), III (96.8), IV (100), A (38.7), NAA (27.5), AA without HGD (47), AA with HGD (62.5), SA (27.8), TA (28.7), TVA (53.7), VA (83.3)		[27]
*SEPT9*	China	465 CRC, 164 A, 108 HPP	610 NED	I (52), II (129), III (165), IV (25), SA (15), TA (84), TVA (54), VA (11)	Plasma	Epi proColon 2.0	CRC versus NED: 0.81, CRC versus A/HPP/NED: 0.8, CRC versus A/HPP: 0.78, CRC IV versus I–III: 0.73, VA versus no VA: 0.59, BL versus NED: 0.51	I (53.8), II (80.6), III (77.4), IV (84.2), HPP (9.3), SA (13.3), TA (19), TVA (41.2), VA (66.7)		[28]
*SEPT9* (10 subregions)	China	53 CRC, 48 AA, 30 BL	48 N	I (14), II (16), III (9), IV (14)	Plasma	MSP	0.882 (< 0.0001)	all (84.9), I (64.3), II (81.3), III (100), IV (100), AA (23), BL (40)	all (83.3)	[29]
*BCAT1*	Australia	74 CRC	144 N	I (4), II (28), III (23), IV (8)	Plasma	MSP		CRC (64.9), I (25), II (64), III (65), IV (88)	96.5	[32]
*IKZF1*								CRC (67.6), I (25), II (57), III (78), IV (100)	95.1	
*BCAT1 and IKZF1*								CRC (77), I (50), II (68), III (87), IV (100)	92.4	
*BCAT1*	Australia, Netherland, Denmark	184 CRC, 337 AA, 279 NAA	820 N	I (41), II (57), III (51), IV (33)	Plasma	MSP	CRC (0.71)	CRC (47.3), AA (8.6), NAA (4.7), I (19.5), II (52.6), III (45.1), IV (75.8)	94.6	[33]
*IKZF1*							CRC (0.775)	CRC (59.2), AA (8.6), NAA (4.3), I (26.8), II (70.2), III (64.7), IV (69.7)	95.7	
*IRF4*							CRC (0.739)	CRC (50), AA (5.9), NAA (2.9), I (17.1), II (57.9), III (54.9), IV (66.7)	97.8	
*BCAT1 and IKZF1*							CRC (0.808)	CRC (70.1)	91.5	
*BCAT1/IKZF1/IRF4*							CRC (0.82)	CRC (73.9), AA (15.7), NAA (9.3), I (39), II (87.7), III (78.4), IV (84.8)	90.1	
*SEPT9*	China	111 CRC	114 NED	I (13), II (49), III (39), IV (7)	Serum	MSP	0.854	all (73), I (38.5), II (81.6), III (69.2), IV (100)	95.6	[16]
*SDC2*							0.881	all (71.2), I (53.9), II (67.4), III (79.5), IV (85.7)	95.6	
*SEPT9 and SDC2*						ColoDefense	0.922	all (86.5), I (69.2), II (85.7), III (89.7), IV (100)	92.1	
*SEPT9*	China	117 CRC, 23 AA	166 N, 78 SP	I (20), II (50), III (38), IV (4)	Plasma	MSP	CRC: 0.9, AA: 0.579	all (82.1), I (65), II (84), III (86.8), IV (100), AA (12.1)	95.8, with SP (92.6)	[17]
*SDC2*							CRC: 0.886, AA: 0.754	all (69.2), I (55), II (74), III (65.8), IV (100), AA (43.5)	95.8, with SP (93.4)	
*SEPT9 and SDC2*						ColoDefense	CRC: 0.941, AA: 0.754	all (88.9), I (80), II (90), III (89.5), IV (100), AA (47.8)	92.8, with SP (87.7)	
*SDC2*	Korea	131 CRC	125 N	Korean: I (11), II (28), III (36), IV (12), European: I (15), II (29)	Serum	MethyLight	0.927 (0.0001)	all (87), I (92.3), II (82.5), III (88.9), IV (91.7)	95.2	[36]
*ALX4*	Germany	30 CRC	30 N	I (4), II (6), III (17), IV (3)	Serum	MethyLight	0.839	83.3	70	[37]
*SFRP1*	Hungary	47 CRC, 37 A	37 N		Plasma	MethyLight	CRC: 0.869, A: 0.824 (< 0.0002)	CRC (80.9), A (89.2)	CRC (83.8), A (73)	[38]
*SFRP2*							CRC: 0.863, A: 0.789 (< 0.0002)	CRC (63.8), A (81.1)	CRC (97.3), A (73)	
*SDC2*							CRC: 0.930, A: 0.859 (< 0.0002)	CRC (87.2), A (73)	CRC (100), A (94.6)	
*PRIMA1*							CRC: 0.822, A: 0.782 (< 0.0002)	CRC (57.4), A (59.5)	CRC (100), A (97.3)	
*SFRP1/SFRP2/SDC2/PRIMA1*							CRC: 0.978, A: 0.937	CRC (91.5), A (89.2)	CRC (97.3), A (86.5)	
*SFRP2*	China	62 CRC, 7 AA	55 N	I (13), II (27), III (17), IV (5)	Serum	MethyLight	0.821	all (69.4), I (46.2), II (74.1), III (70.6), IV (100), AA (42.9)	87.3	[39]
*OSMR*	Italy	70 CRC	18 A, 36 N	I (11), II (15), III (29), IV (15)	Plasma	MethyLight	CRC versus N: 0.6944, CRC versus A: 0.6472 (0.002)	CRC versus NorA (44.3)	CRC versus N (86.1), CRC versus A (83.3)	[42]
*SFRP1*							CRC versus N: 0.7952, CRC versus A: 0.7560 (< 0.0001)	CRC versus NorA (62.9)	CRC versus N (91.7), CRC versus A (83.3)	
*VIM*	Germany	81 CRC	110 N		Plasma	Methyl-BEAMing	all: 0.81, Duke's A: 0.77, B: 0.82, C: 0.67, D: 0.95	all S (59), Duke's A&B (52)	93	[43]
*B4GALT1*	Italy	TC: 20 CRC, VC: 26 CRC	19 N		Plasma	dd-QMSP	TC: 0.750 (0.008)	TC: 50	TC: 100	[45]
*WIF1*	Korea	243 CRC, 64 A	276 N	I (44), II (199)	Plasma	MSP	0.641	36.7	90.6	[46]
*APC, MGMT, RASSF2A, WIF1*							CRC: 0.927, A: 0.864	CRC (86.5), A (74.6)	CRC (92.1), A (91.3)	
*JAM3 or JAMC*	China	18 CRC	18 N	I (1), II (4), III (7), IV (3)	Plasma	MSP	0.8611 (< 0.001)			[47]
*PCDH18*	China	20 CRC	20 N		Plasma	MSP	0.85 (< 0.05)			[48]
*NEUROG1*	Germany	45 CRC	16 N	UICC I (11), II (9), III (7), IV (18) (marker comparison set)	Serum	MethyLight		55.5	81.3	[49]
*ALX4*								46.6	66.3	
*SEPT9*								46.6	81.3	
*VIM*								41.1	60	
*NEUROG1*		92 CRC	45 N	UICC I (27), II (70) (test set)			0.73 (< 0.0001)	UICC I (51.9), II (64.3)	91.1	
*NDRG4*	Germany	TC: 154 CRC, VC: 66 CRC	TC: 444 N, VC: 240 N	TC: I (43), II (44), III (46), IV (21), VC: I (27), II (15), III (20), IV (4)	Plasma	MSP	TC: 0.61	TC: all (27), I (16), II (11), III (35), IV (62)	TC (95)	[50]
*GATA5*							TC: 0.59	TC: all (18), I (14), II (9), III (17), IV (47)	TC (99)	
*FOXE1*							TC: 0.7	TC: all (46), I (35), II (43), III (50), IV (67)	TC (93)	
*SYNE1*							TC: 0.72	TC: all (47), I (28), II (52), III (46), IV (76)	TC (96)	
*SYNE1 and FOXE1*								TC: all (56), I (42), II (57), III (59), IV (81), VC: all (58), I (37), II (87), III (55), IV (100)	TC (90), VC (91)	
*C9orf50*	Netherlands	75 CRC	66 N	I (19), II (24), III (31), IV (1)	Plasma and serum	Digital MethyLight	plasma: 0.7, serum: 0.69			[51]
*THBD*							plasma: 0.8, serum: 0.82	plasma (71)	plasma (80)	
*C9orf50 and THBD*							plasma: 0.8, serum: 0.83			
*HIC*	Italy	30 CRC	30 N	I (11), II (19)	Plasma	MSRE-PCR	0.858	76.67	83.3	[52]
*CYCD2*							0.8322	70	73.3	
*VHL*							0.703	75.8	66.67	
*HIC/CYCD2/VHL*		30 CRC + VC: 10 CRC	30 N + VC: 10 N				0.9379, VC: 0.9	82.76, VC (70)	93.3, VC (90)	
*LINE-1*	Japan	114 CRC	53 N	I–II (57), III–IV (57)	Plasma	AQAMA-real time PCR	all: 0.81, I–II: 0.79, III–IV: 0.83 (< 0.0001)	all (65.8), I–II (63.2), III–IV (68.4)	all (90), I–II (90), III–IV (90)	[53]
*ALX4, BMP3, NPTX2, RARB, SDC2, SEPT9, and VIM*	Denmark	193 CRC	102 N including 33 with resectable A		Plasma	MSP	all: 0.887, I–II: 0.8775 (= 0.3512)	all (90.7), I–II (88.7)	all (72.5), I–II (73.5)	[13]
80 markers	China	TC: 73 CRC	TC: 70 N	I (18), II (34), III (20), IV (1)	Plasma	MCTA-Seq	0.88	all (75), I (65), II (76), III–IV (81)	94	[56]
		VC: 74 CRC	VC: 66 N	I (14), II (33), III (23), IV (4)			0.89	all (79), I (62), II (81), III–IV (85)	86	
		CC: 147 CRC	CC: 136 N	I (32), II (67), III (43), IV (5)				I–II (74)	I–II (90)	
9 markers*	China	TC: 528 CRC	TC: 674 N	I (38), II (139), III (209), IV (406)	Plasma	Deep sequencing	9 markers: 0.96, cg10673833: 0.904	87.5	89.9	[57]
		VC: 273 CRC	VC: 347 N				9 markers: 0.96, cg10673833: 0.91	87.9	89.6	
cg10673833		29 CRC, 78 APL, 114 NAA, 250 BL	1021 N			dd-PCR	0.9	CRC (89.7), APL (33.3), NAA (21.9), BL (8)	CRC (86.8), APL (66.7), NAA (78.1), BL (92)	
CpG sites	China	TC: 149 CRC, VC: 67	TC: 149 N, VC: 74	TC: I (34), II (54), III (35), IV (26), VC: I (17), II (25), III (15), IV (10)	Plasma	MSP	TC: 0.943, VC: 0.934	TC: CRC (88.6), I (79.4), II (88.9), III (91.4), IV (96.2), I–III (87), VC: CRC (83.6), I (70.6), II (88), III (86.7), IV (90), I–III (83.5), TC + VC: CRC (87), I (76.5), II (88.6), III (90), IV (94.4)	TC (89.3), VC (91.9), TC + VC (90.1)	[58]
*SEPT9*							TC: 0.655, VC: 0.673	TC + VC: CRC (41.2), I (19.6), II (36.7), III (46), IV (75)	TC + VC (90.6)	
13 markers ***	Minnesota, USA	97 CRC	200 N	I (11), II (26), III (24), IV (23)	Plasma	TELQAS	all: 0.91	all (77), I (64), II (65), III (71), IV (100)	95	[49]
*NPY, PENK, WIF1*	France	32 CRC, 26 P	161 N	I–II (6), III–IV (26)	Serum	QM-MSP		CMI 0.62–0.85 (87)	CMI 0.62–0.85 (80)	[60]
								CMI 0.94 (78)	CMI 0.94 (90)	
								CMI 2.01 (59)	CMI 2.01 (95)	
5 markers **	Korea	97 CRC	60 N	I (17), II (24), III (33), IV (23)	Plasma	ddMethyLight		I–III (45.9), IV (95.7)	95	[61]

^*^(cg16959747, cg10673833, cg21939215, cg24067911, cg17494199, cg23678254, cg10428836, cg10493436, cg25459300), **(FAM123A, GLI3, PPP1R16B, SLIT3, TMEM90B), ***(FER1L4, VAV3, CHST2, DTX1, PDGFD, SFMBT2, QKI, ZNF568, ANKRD13B, ZNF671, CNNM1, GRIN2D, JAM3)

A, adenoma; AA, advanced adenoma; APL, advanced precancerous lesions; AQAMA, absolute quantitaive analysis of methylates alleles; AUC, area under curve; BL, benign lesions; CC, combined cohort; CMI, cumulative methylation index; CRC, colorectal cancer; ddMethyLight, droplet digital MethyLight; dd-PCR, droplet digital PCR; dd-QMSP, droplet digital quantitative methylation-specific PCR; GID, gastrointestinal diseases; HGD, high grade dysplasia; HM, heavy methyl quantitative real-time PCR; HPP, hyperplastic polyps; HRA, high-risk adenoma; INF, inflammation; LRA, low-risk adenoma; MCTA-Seq, methylated CpG tandem amplification and sequencing; MSRE-PCR, methylation sensitive restriction enzyme and multiplex PCR; NED, no evidence of disease; NAA, non-advanced adenomas; N, normal; PL, precancerous lesions; P, polyps; QM-MSP, quantitative multiplex methylation-specific PCR; SA, serrated adenoma; SSP, serrated sessile adenomas/polyps; SP, small polyps; TELQAS, target enrichment long-probe quantitative-amplified signal TA, tubular adenoma; TVA, tubulovillous adenoma; VA, villous adenoma; TC, test cohort; VC, validation cohort

So far, Epi proColon, with its improved edition Epi proColon 2.0, is the only blood-based DNA hypermethylation screening test for CRC that has been approved by the Food and Drug Administration (FDA) [13]. This test is based on a qualitative real-time polymerase chain reaction (PCR) detection of methylated *Septin 9* (m*SEPT9*) DNA. *SEPT9* is a member of the septin gene family, a group of GTP binding protein that was first identified in yeast as key regulators of cell division. Although its role in colorectal cancer development is still not well understood, hypermethylation of *SEPT9* has been reported extensively in CRC patients [14, 15]. Other tests have also been utilized to analyze the methylation status of *SEPT9* as an individual marker or in combination with other markers. These include ColoDefense, MethyLight, and Heavy MethyLight assays that rely on the quantitative methylation-specific real-time PCR (qMSP) detection. Sensitivity of the analyzed m*SEPT9* in plasma/serum ranged from 47 to 87% with a specificity ranging from 89 to 98% in a wide variety of studies [13, 16–28]. Sensitivity of m*SEPT9* gradually increased with higher stages and was reported to be 100% in stage IV CRC patients in some studies, however, remaining low in early stages (I–II) [16, 17, 22, 27, 28]. Notably, *SEPT9* methylation showed higher sensitivity in diagnosing CRC than the conventional markers CEA and CA-19-9, or even FOBT [18, 19, 25], and when combining m*SEPT9* with either one of them, diagnostic sensitivity, especially for early stages, increased [18, 25, 26]. Furthermore, sensitivity of m*SEPT9* for the detection of adenomas and polyps was relatively low in most studies, ranging from 8 to 40% [17–19, 21, 23, 24, 28]. Most of these studies used the 1/3 algorithm, which means that the sample is considered positive if one of three PCR replicates is positive. However, a recent study by Song et al. reported a high positive detection rate (PDR) in villous adenoma and adenoma with high-grade dysplasia (83.3% and 62.5%, respectively), although the rate for all adenomas was much lower (31.8%) [27]. In this study, they used the 2/3 algorithm, which might explain the difference in the obtained results. A recent study detected 10 different methylation subregions within the *SEPT9* gene at a sensitivity of 84.9% and a specificity of 83.3% (Area under the curve, AUC = 0.882) in a cohort of 53 CRC patients, 48 patients with AA, 30 patients with benign polyps, and 48 healthy controls. To compare whether this multi-marker approach produced better detection of early-stage CRC and precancerous lesions from the usual single-marker approach that is commercially used, a new cohort of 43 CRC patients, 15 patients with AA, 15 patients with benign polyps, and 30 controls was recruited. Both approaches had the same high specificity (90%). When compared with the usual single-marker approach, the sensitivity of the multi-marker approach was higher for early-stage CRC (73.3% vs. 60% for stage I, 76.5% vs. 70.6% for stage II) and was statistically higher for AA and polyps (53.3% vs. 26.7% for AA, 33.3% vs. 6.7% for polyps) [29].

Other defined markers for CRC detection described in more than one study include methylated *BCAT1* (branched-chain amino acid transaminase 1), *IKZF1* (IKAROS family zinc finger 1), *SDC2* (syndecan-2), *ALX4* (aristaless-like homeobox 4), *SFRP2* (secreted frizzled-related protein 2), *OSMR* (oncostatin M receptor), *SFRP1* (secreted frizzled-related protein 1), and *VIM* (vimentin). Methylation of *BCAT1* and *IKZF1* is regularly described together*. BCAT1* gene encodes an enzyme involved in catabolism of branched-chain amino acids and *IKZF1* gene encodes a transcription factor that regulates a small set of cell cycle-regulator genes [30, 31]. Sensitivity of *BCAT1* ranged between 47.3 and 64.9% and that of *IKZF1* ranged between 48 and 67.6% for CRC detection. In addition, the positivity rates increased with higher CRC stages but were very low for adenomas [32–34]. Another reported gene, *SDC2,* promotes cell proliferation, migration, and invasion, inhibits apoptosis, and activates epithelial to mesenchymal transition (EMT) and mitogen-activated protein kinase (MAPK) signaling pathways in CRC cells [35]. *SDC2* might be a highly promising methylation marker for CRC detection where it displayed a sensitivity that ranged between 69 and 87% for all stages and a specificity of approximately 95% in the plasma/serum of CRC patients. Recent studies showed that the PDR of *SDC2* methylation test increased with higher tumor stage, ranging between 55 and 100% for stages I–IV [16, 17, 36]. In addition, methylation of *ALX4*, a transcription factor involved in limb and skull development, was more frequently found in the serum of CRC patients compared to normal individuals (sensitivity of 83.3%, specificity of 70%, AUC = 0.839) [37]. Methylation of another gene, *SFRP2,* a member of the SFRP family that act as soluble modulators of Wnt signaling, is also reported as a potential marker for CRC detection. *SFRP2* methylation analysis showed a sensitivity of 63.8/69.4% for CRC detection and 42.9%/81.8% for adenoma detection [38, 39]. Compared to *SEPT9*, sensitivity of *SFRP2* for AA detection is much higher suggesting that *SFRP2* might be a viable biomarker for the detection of precancerous lesions. *OSMR* is reported as a tumor suppressor in colon cancer progression; promoter methylation correlated with loss of *OSMR* expression in CRC cells and low expression of *OSMR* was associated with resistance to growth inhibition [40]. Another gene, *SFRP1*, is a tumor suppressor gene that inhibits cell proliferation, migration, and invasion, and mediates apoptosis of CRC cells [41]. Methylation of *OSMR* and *SFRP1* genes was shown to be significantly higher in CRC and adenoma than in normal plasma samples [21, 42]. Furthermore, *VIM* gene, a target of epigenetic modifications, is frequently methylated in CRC. *VIM* methylation was shown to be higher in CRC plasma compared to normal samples with a sensitivity of 59% and specificity of 93% (AUC = 0.81) [43]. All mentioned markers are reported to be methylated in CRC tissues, blood, and/or stools [44]. Overall, these markers showed promising results in the detection of CRC and thus require further validation in larger cohorts. Other less frequently described candidate markers that require further investigation and validation include B4GALT1 [45], *WIF1* [46], *JAM3* [47], *EYA4* and *TAC1* [20], *PCDH18* [48], *NEUROG1* [49], *TMEFF2* and *NGFR* [22], *NDRG4, GATA5*, *FOXE1*, and *SYNE1* [50], *C9orf50* and *THBD* [51], *HIC*, *CYCD2*, and *VHL* [52], and hypomethylated *LINE-1* [53].

Several studies investigated the diagnostic performance of a panel of methylated genes and showed that the simultaneous analysis of multiple hypermethylated circulating DNA is more sensitive in detecting CRC than the individual markers. Multiple studies showed that a panel of methylated genes containing m*SEPT9* might be promising in the detection of precancerous lesions and early-stage CRC. The simultaneous analysis of methylated *SEPT9* and *SDC2* increased the sensitivity for all stages and for each stage without significant effect on specificity [16, 17]. In addition, sensitivity of AA detection was higher for the combinatorial markers (47.8%) than for each marker individually (12.1% for *SEPT9* and 43.5% for *SDC2*) [17]. These studies have shown that methylated *SEPT9* and *SDC2* panel might be one of the best combinations for early CRC screening. Analysis of *BCAT1* and *IKZF1*, simultaneously, showed a sensitivity of 77% for CRC at a specificity of 92.4%. This model improved the detection rate of CRC with a small decrease in the specificity (*BCAT1* sensitivity: 64.9%, specificity: 96.5%; *IKZF1* sensitivity: 67.6%, specificity: 95.1%) [32]. A recent study reported the test performance of different methylation target combinations (*BCAT1/IKZF1, BCAT1/IRF4* (Interferon regulatory factor 4)*, IKZF1/IRF4,* and *BCAT1/IKZF1/IRF4)* and showed that the best sensitivity (73.9%) was achieved using the three gene markers (*BCAT1/IKZF1/IRF4)* with a specificity of 90.1% and an AUC of 0.82. In addition, the positivity rates for detecting AA and non-advanced adenoma (NAA) were the highest in this panel although remaining very low (15.7% for AA, 9.3% for NAA) [33]. However, more than one study showed that the use of a single positive PCR replicate for methylated *BCAT1* can yield false positive results [33, 54]. To overcome this, Young et *al.* used a “*BCAT1* replicate rule” that states that a specimen is positive if at least two PCR replicates were positive for *BCAT1* (along with at least one positive PCR replicate for *IKZF1* or *IRF4)*. In this way, the false-positive rate for CRC significantly decreased from 9.9 to 5.9% without any significant effect on sensitivity, but it significantly decreased the sensitivity of AA detection from 15.7 to 11% [33]. Furthermore, age (≥ 70 years) and cell free DNA yield were significant independent factors associated with the detection of methylated *BCAT1/IKZF1* in the patients with no CRC [54]. Another study showed low sensitivity of *BCAT1/IKZF1* in detecting sessile serrated adenomas/polyps (SSP) (8.8%) and when combined with FIT, it remained low (26.5%) but still higher than FIT alone (16.3%) [55].

The combined analysis of *ALX4*, *BMP3* (bone morphogenetic protein 3), *NPTX2* (neuronal pentraxin 2), *RARB* (retinoic acid receptor beta), *SDC2*, *SEPT9*, and *VIM* displayed a sensitivity of 90.7% for all CRC stages and 88.7% for stages I/II using a model accounting for the covariates female gender and age greater than 66 years however, with a *p* value > 0.05 [13]. This logistic regression model was considered the most applicable among 17 developed models in the study since it contained a limited number of genes and it did not differ from the model produced by another method which is Penalized regression using Firth’s method. Interestingly, in this study, they showed the low sensitivity to some individual markers, reiterating the importance of a panel of genes as diagnostic biomarkers [13]. Moreover, Tanzer et al*.* proved that the combined analysis of methylated *SEPT9* and *ALX4* was highly significant in the detection of advanced precancerous colorectal lesions with a 71% sensitivity and a 95% specificity. This study presents an approach for the detection of precancerous lesions using methylated markers in plasma of CRC patients; however, it should be validated on a larger cohort [23]. In addition, a panel of 80 hypermethylated markers detected by methylated CpG tandem amplification and sequencing (MCTA-Seq) method distinguished early-stage CRC patients from normal individuals with 74% clinical sensitivity and 90% specificity. These markers included known ones like *SEPT9* and *IKZF1* and novel ones including *TJP2* (tight junction protein 2) and *GATM* (glycine amidinotransferase, mitochondrial) [56]. Another study distinguished CRC from normal controls through 9 methylated markers with 87.5% sensitivity and 89.9% specificity (AUC = 0.96) in the training cohort, and 87.9% sensitivity and 89.6% specificity (AUC = 0.96) in the validation cohort, by constructing a combined diagnostic score (cd-score). They then showed that cg10673833 displayed the best diagnostic performance with AUC of 0.904 and 0.91 for training and validation cohort, respectively [57]. Moreover, *Sui *et al*.* reported that the methylation of specific CpG sites in plasma can be used as an early CRC detection model. The selected CpG sites were based on enrichment of CRC-related methylated variation signal, based on the 450 K microarray data of CRC samples, normal samples and white blood cells from The Cancer Genome Atlas (TCGA) and Gene Expression Omnibus (GEO) datasets. These ctDNA methylation markers had a sensitivity of 88.6% and a specificity of 89.3% (AUC = 0.943) in a training set composed of 149 CRC and 149 healthy controls, and a sensitivity of 83.6% and a specificity of 91.9% (AUC = 0.934) in the test set composed of 67 CRC and 74 healthy controls. The sensitivity increased with higher CRC stages in both the training and test sets. The performance of the model in the unmatched population was similar to that of the matched population in detecting the different CRC stages. In addition, this model had higher sensitivity when compared to *SEPT9* model (41.2%) with a comparable specificity making it a more promising approach in the early detection of CRC [58]. In addition, a panel of 13 methylated DNA markers (*FER1L4*, *VAV3, CHST2, DTX1, PDGFD, SMBT2, QKI, ZNF568, ANKRD13B, ZNF671, CNNM1, GRN2D, and JAM3*), in the plasma of 97 CRC and 200 controls, detected all stages of CRC with a sensitivity of 77%, a specificity of 95%, and AUC of 0.91, higher than that of each marker and than that of CEA. Upon adding CEA to the panel, AUC did not improve [59]. Moreover, a panel consisting of methylated *SFRP1, SFRP2, SDC2,* and *PRIMA1* (proline-rich membrane anchor 1) genes might allow noninvasive detection of colorectal adenoma and cancer from plasma samples, where they displayed higher sensitivities than that of the individual genes. This panel distinguished CRC patients (n = 47) from normal ones (n = 37) with a 91.5% sensitivity and a 97.3% specificity (AUC = 0.978), and adenoma samples (n = 37) with a sensitivity of 89.2% and specificity of 86.5% (AUC = 0.937) [38]. Other described panels that might be valuable diagnostic biomarkers for CRC detection include *SEPT9* and *OSMR* [21], *SEPT9* and *TAC1* [20], *HIC/CYCD2/VHL* [52], *APC/MGMT/RASSF2A/WIF1* [46], *SYNE1* and *FOXE1* [50], *WIF1/NPY/PENK* [60], *THBD* and *C9orf50* [51], and *FAM123A/GLI3/PPP1R16B/SLIT3/TMEM90B* [61]. Overall, these studies have shown that the use of marker panels is of high accuracy in the detection of CRC and is more promising than the use of single markers.

Most of the studies analyzed methylation status of the genes in CRC tissues by microarray analysis, Illumina methylation array, or MSP then, validated the results in the plasma and/or serum by MSP dependent assays. Several studies reported different sensitivities/specificities of the same diagnostic methylated marker. Differences in marker performance might be due to differences in ethnicity, choice of control population, sample type, or DNA extraction methods. Other frequently reported markers include *APC, CDKN2A, HLTF, MLH1, HPP1, RUNX3,* and *SHOX2*, and less frequently described markers include *TMEM240, AKAP12, BNC1, BRCA1, CDH1, CDH4, CRABP1, DAPK1, DLC1, ERCC1, FBN2, FGF2, FHIT, GRASP, IRF4, ITGA4, LRR3CB, MAL, NELL1, PCDH10, PDX1, PHACTR3, PPENK, RASSF1A, SMAD4, SOX21, SPG20, SST, TFPI1,* and *WNT5A,* some of which showed high sensitivities, therefore, supporting their further investigation and validation [62]. These markers, along with the previously described ones, were investigated in multiple studies, but we did not include them since ROC curves were not established to validate their diagnostic performance.

### DNA methylation as a biomarker for CRC prognosis and prediction

DNA methylation has been also explored as a potential biomarker for CRC prognosis and therapy prediction whereby it can predict the overall cancer outcome and recurrence of the disease as well as the effectiveness of a treatment. Most of current CRC prognostic and predictive biomarkers are mainly tissue derived and vary due to intratumoral heterogeneity as well as heterogeneity between metastases. Increased prognostic severity of CRC has been identified with increase in tumor tissue staging (TNM staging), presence of *BRAF* mutation especially V600 mutation [63], microsatellite stability due to activation of the mismatch repair genes as compared to microsatellite instability [64], and presence of mutations in *SMAD4* and *APC* genes [65, 66]. However, analysis of multiple biopsies is not feasible in the clinical routine, and this is unsuitable for neoadjuvant treatment decisions which makes these markers inefficient. Computed tomography is a method for disease assessment, but it cannot be used routinely since this radiation method cannot be repeated frequently as an instant test.

As for blood markers, higher ctDNA levels (especially *KRAS*, *APC* and *TP53* mutations) have been reported with poor outcome [67, 68] and elevated neutrophil-to-lymphocyte ratio has been associated with short overall survival (OS) and progression-free survival (PFS) after treatment in CRC patients [69, 70]. However, CEA is the main current serum marker to assess recurrence especially every 3 months post-surgery for CRC patients with stage II or III [71] or to have intensive follow-up every 3–6 months for CRC along with CT every 3–12 months [72]. CEA is more sensitive to advanced stage CRC than early stage CRC, which restricts its use for many surgical patients [73]. Despite these examinations, novel prognostic, and predictive biomarkers such as methylated ctDNA that are easily detected are needed (Table 2).

**Table 2:** Prognostic and predictive methylated ctDNA in colorectal cancer patients

Gene	Country	Type of study	No. of cases	Time of sample collection	Cases characteristics	FU duration	Sample type	Technology used	Hypermethylation reported association with	HR (*p*-value)	References
*HPP1*	Germany	Prospective	77	Pretherapy	UICC I (10), II (24) III (27), IV (15)	5 Y	Serum	MethyLight	Higher risk of death	5.1 (0.001)	[77]
*HLTF*										3 (0.008)	
*HPP1 and/or HLTF*										HR (uni) = 4.2 (< 0.001), HR (multi) = 3.4 (0.007)	
*HLTF*	Germany	Prospective	103	Pretherapy	UICC IV	6 Y	Serum	MSP	Worse OS in stage IV	1.8 (0.0438)	[76]
*HPP1*	Germany	Prospective	103	Pretherapy	UICC IV	6 Y	Serum	MSP	Worse OS in stage IV	1.6 (0.0495)	[76]
*HLTF*	Germany	Prospective	259	Pretherapy	I (51), II (68), III (51), IV (89)	10 Y	Serum	MethyLight	Shorter OS (*p* = 0.0008) esp. in stage IV (*p* = 0.0081)		[78]
*HPP1*	Germany	Prospective	259	Pretherapy	I (51), II (68), III (51), IV (89)	10 Y	Serum	MethyLight	Shorter OS (*p* < 0.0001) esp. in stage IV (*p* = 0.0005)		[78]
*HPP1*	Germany	Prospective	467	Pretherapy and after first cycle	mCRC on combination of a fluoropyrimidine, oxaliplatin and bevacizumab	24 W	Plasma	MethyLight	At baseline with worse OS, after the first cycle with high risk of progression	HR baseline = 1.86 (0.0001), HR during treatment = 2.13 (0.0001)	[79]
*HLTF*	Germany		106	Pretherapy	UICC I–III	5 Y	Serum	MethyLight	Increased risk of recurrence	HRuni = 2.7 (0.014), HRmulti = 2.5 (0.023)	[80]
*SEPT9*	Singapore	Prospective	150	Preoprative, 6 M-FU and 1Y-FU	I–III; 1 neoadjuvant treatment and 45 adjuvant chemo and radiotherapy	7 Y	Serum	MSP	1Y-FU with poor DFS and CSS; dynamic change from 6 M to 1Y and from baseline and 1Y with recurrence	HR (1Y-FU; CSS) = 2.69 (< 0.05); HR (1Y-FU;DFS) = 3.50 (0.001); HR (6 M-FU to 1Y-FU;DFS) = 2.58 (0.05), HR (baseline to 1Y-FU;DFS) = 3.35 (0.01)	[81]
*SEPT9*	China	Prospective	98	Preoperative and at 3 M intervals	Performed surgery	28 M	Plasma	MSP	Postoperative with higher mortality rate (*p* = 0.024) and presence of mets (*p* = 0.013) and lower OS (*p* = 0.014)		[18]
*SEPT9*	China	Retrospective	300 from china, 330 from TCGA	Preoperative	Absent	30 M for Chinese people, 125 M for TCGA	Plasma	MSP	Shorter PFS (*p* = 0.019) and OS (*p* = 0.008)		[83]
*SEPT9*	China	Prospective	82	Preoperative and 1 and 7 days postoperative	I (14), II (40), III (45)	21 M	Plasma	MSP	Higher risk of death post-surgery	HR (OS) = 2.51 (0.036)	[82]
*SEPT9* (10 subregions)	China	Prospective	82	Postoperative (within 2 W)		3Y	Plasma	MSP	Poor RFS	HR (RFS) = 4.20 (0.0005)	[29]
*SEPT9* (10 subregions)	China	Prospective	19	Serial postoperative		3Y	Plasma	MSP	Poor RFS; better in recurrence prediction than single detection	HR (RFS) = 7.49 (0.01)	[29]
*NPY*	Denmark	Prospective	97	Pretherapy, 2 W of treatment and before every new cycle	mCRC receiving regorafenib as last-line treatment	Every second week for 2 months and then monthly if stable	Plasma	MSP ddPCR	Baseline with shorter OS (*p* < 0.001)		[86]
*NPY*	Denmark and Canada	Prospective	146	Pretherapy	Locally advanced rectal cancer taking Neoadjuvant Chemoradiotherapy	5 Y	Serum	MSP ddPCR	Higher risk of death and distant disease progression	HR (OS) = 2.08 (0.007), HR (distant mets) = 2.20 (0.01)	[88]
*NPY*	Denmark	Prospective	123	Pretherapy	mCRC taking 5-FU, oxaliplatin, and bevacizumab	7.5 Y	Plasma	MSP ddPCR	Shorter PFS and OS	HR (PFS) = 0.48 (0.0005), HR (OS) = 0.50 (0.0001)	[89]
*RASSF1A*	Greece	Prospective	155	Preoperative	Early operable (88), mets (67)	8 Y	Serum	MSP	Worse survival in early and mets; more pronounced in mets	HR (early; OS) = 3.06 (0.038), HR (mets; OS) = 5.76 (0.001)	[93]
*RASSF1A*	China	Prospective	108 CRC, 78 healthy	Pretherapy and after two cycles	II–III receiving oxaliplatin-based chemo	3 Y	Blood	MSP	Shorter PFS and OS	HR = 2.471 (0.02)	[92]
*BCAT1 and IKZF1*	Australia and New Zealand	Prospective	172	12 M post-surgery	Invasive CRC requiring surgery	12 M	Plasma	MSP	Increased risk of residual disease and recurrence	HR = 3.8 (0.004)	[94]
*BCAT1 and IKZF1*	USA	Prospective	322	Within 6 M post-therapy	Stage II or III CRC		Plasma	MSP (COLVERA)	Increased recurrence		[97]
*BCAT1 and IKZF2*	Australia and New Zealand	Prospective	144	Within 12 M in remission	I (21), II (50), III (62), IV (11)		Plasma	MSP (COLVERA)	Increased recurrence		[96]
*BCAT1 and IKZF3*	Australia and New Zealand	Prospective	122	3, 6, or 12 M in remission	I (28), II (40), III (47), IV (4)		Plasma	MSP	Increased recurrence		[95]
*SST*	Singapore	Prospective	165	Preoperative	Done surgery without neoadjuvent chemo	7 Y	Serum	MSP	Higher risk of cancer-specific death esp stage III and risk of recurrence	HR (OS) = 1.96 (0.031), HR (DFS) = 2.60 (0.003)	[99]
*TAC1*	Singapore	Prospective	150	Preoperative, 6 M-FU and 1Y-FU	I–III; 1 neoadjuvant treatment and 45 adjuvant chemo- and radiotherapy	7 Y	Serum	MSP	6 M-FU with poor DFS and CSS; dynamic change from baseline to 6 M with recurrence	HR (6 M-FU; CSS) = 4.12 (< 0.001), HR (6 M-FU, DFS) = 5.72 (< 0.001), HR (from baseline to 6 M-FU; DFS) = 4.71 (< 0.001)	[81]
*APC*	Greece	Prospective	155	Preoperative	Early operable (88), mets (67)	8 Y	serum	MSP	Worse survival in early and mets; more pronounced in early operable	HR (early; OS) = 7.88 (< 0.001), HR (mets; OS) = 3.47 (0.017)	[93]
13 markers ***	Minnesota, USA	Prospective	40 recurrent, 60 healthy	Post-surgery	I (11), II (26), III (24), IV (23)		Plasma	TELQAS	detect recurrent/metastatic CRC with 90% sensitivity, 90% specificity, AUC = 0.96		[59]
*MYO1G, CALML4, GCET2, KLF3, ATXN1*	China	Prospective	528	Pretherapy	Training cohort	26.6 M	Plasma	Deep sequencing of bis-DNA	High cp-score associated with poor prognosis (OS)	2.24 (< 0.001)	[57]
			273		Validation cohort	26.6 M				2.21 (< 0.001)	
*EYA4, GRIA4, ITGA4, MAP3K14-AS1, MSC*	Italy	Retrospective	60 before and 62 during treatment	Pretherapy and biweekly during regorafenib treatment	mCRC patients who received regorafenib	5.5 M (1.25–56.5 M)	Plasma	Methyl-BEAMing	Baseline with worse OS and shorter PFS, during treatment with shorter PFS	HR baseline (OS) = 3.471 (0.0001), HR baseline (PFS) = 2.196 (0.0015), HR during treatment (PFS) = 2.985 (< 0.0001), HR dynamic (PFS) = 1.78 (0.028)	[101]

^***^(FER1L4, VAV3, CHST2, DTX1, PDGFD, SFMBT2, QKI, ZNF568, ANKRD13B, ZNF671, CNNM1, GRIN2D, JAM3)

CECT, contrast enhanced computed tomography; Chemo, Chemotherapy; CSS, Cancer-specific survival; ddPCR, digital droplet PCR; DFS, Disease-free survival; FU, fluorouracil; MethyLight, fluorescence-based real time PCR; Mets, Mestastasis; M, month; MS-HRM, methylation‐sensitive high‐resolution melting assay; Multi, Multivariate; OS, Overall Survival; PFS, Progression-free survival; RFS, recurrence-free survival; TELQAS, target enrichment long-probe quantitative-amplified signal TA, tubular adenoma; 1FU, 1-year follow-up; 6MFU, 6-month follow-up; Uni, univariate; Y, year

The *HPP1* gene (hyperplastic polyposis 1) encodes a transmembrane protein that is frequently methylated in colorectal tumors [74]. Another gene *HLTF* (helicase-like transcription factor) encodes for a SWI/SNF family protein with both helicase and E3 ubiquitin ligase activities and is also common target for methylation and epigenetic gene silencing in colon cancer [75]. Both methylated ctDNAs of these genes were extensively studied prospectively pretherapy in the serum of CRC patients in several cohorts. They were associated with low OS especially in stage IV CRC [76, 77]. In another study, methylation of *HLTF* and *HPP1* in serum were significantly correlated not only with more advanced stages of CRC but also with high levels of lactate dehydrogenase (LDH) release as a surrogate marker for cell damage [78]. Moreover, plasma levels of methylated *HPP1* (m*HPP1*) ctDNA in a large cohort of metastatic CRC patients was detected before treatment with a combination therapy containing a fluoropyrimidine, oxaliplatin and bevacizumab and then became undetectable after 2–3 weeks of therapy [79]. The baseline level of m*HPP1* ctDNA correlates with poor OS, while its low level after the first treatment correlated with reduced risk of progression. m*HPP1* ctDNA differentiates between responders and non-responders to therapy as determined by the radiological staging after 12 or 24 weeks (AUC = 0.77 or 0.71, respectively). Hence, m*HPP1* ctDNA might be a predictive biomarker for monitoring response to first-line therapy and switching therapy protocols even before doing radiological staging. Furthermore, methylated *HLTF* ctDNA in pretherapy sera of 106 patients curatively resected for CRC were associated with poor outcome and a relative risk of disease recurrence [80]. Hence, it was concluded as a predictor of disease recurrence in CRC even though with Philipp et al*.* 2012, m*HLTF* ctDNA failed to detect identify high risk groups in the UICC II and III subgroups [76].

Methylated *SEPT9* (m*SEPT9*) ctDNA has been also discussed as a prognostic and predictive CRC biomarker. High levels of m*SEPT9* ctDNA is a prominent biomarker for CRC recurrence as shown by more than one study [19, 81]. Elevated level m*SEPT9* detected in postoperative sera of CRC patients (stages I–III) after one year follow-up and its dynamic change from one week before surgery to last follow-up were found to be an independent predictor of tumor recurrence [81]. m*SEPT9* was even a better biomarker for recurrence than CEA where its level at one year showed an earlier lead time advantage of more than 2 months compared to concurrent serum CEA. The combined detection of m*SEPT9* and contrast enhanced CT enhanced the sensitivity (positive detection rate for both is 95.2%) for recurrence monitoring in CRC after radical surgical resection [19]. Furthermore, hypermethylation of *SEPT9* in the plasma of postoperative CRC patients was associated with lower OS and its dynamic increment after surgery correlated with a higher mortality rate and the presence of metastasis [18]. A recent study on m*SEPT9* that used multiple probes for 10 selected subregions of *SEPT9* revealed that positive detection of these markers in postoperative (within 2 weeks) plasma of CRC patients associate with poorer recurrence-free survival. The capacity of these m*SEPT9* markers to predict recurrence did not change upon stratifying the patients according their use of adjuvant chemotherapy but it was affected when dividing patients according to stage (localized, stage II, or stage III CRC) [29]. Detection of positive m*SEPT9* ctDNA in the plasma of CRC patients even at baseline before surgery was correlated with higher risk of death after surgery and shorter PFS and OS [82, 83].

In addition, *NPY* gene (neuropeptide Y) is methylated at high frequency in CRC [84] and it encodes a neuropeptide involved in cell motion and proliferation in CRC [85]. High baseline levels of methylated *NPY* (m*NPY*) ctDNA in plasma of metastatic CRC patients before treatment with regorafenib, oral multi-kinase inhibitor, was correlated with shorter OS. Its measurement was shown to be better even than measuring mutated *RAS*/*RAF* ctDNA since it could be measured in almost all patients irrespective of mutational status [86]. Changes in the longitudinal levels of m*NPY* ctDNA in these metastatic CRC may predict early effect and later progression which is in line with Garrigou et al*.* who was the first to analyze the hypermethylation of *NPY* ctDNA in different stages of CRC in a follow up setting [87]. A recent study revealed that high levels of m*NPY* ctDNA in pretherapy serum samples taken from locally advanced rectal cancer patients was correlated with higher risk of death and distant disease [88]. Additionally, elevated levels of m*NPY* in plasma of metastatic CRC patients before treatment with 5-fluorouracil, oxaliplatin, and bevacizumab was also correlated with shorter OS and PFS [89]. Another studied methylated ctDNA is that of *RASSF1A* gene (RAS association domain family protein 1) which is a tumor suppressor thought to regulate cell proliferation and apoptosis and its expression is reported to be lost in CRC mainly by hypermethylation [90, 91]. Promoter methylation of *RASSF1A* in blood was reported to be a prognostic biomarker for patients with stage II and III CRC treated with oxaliplatin-based chemotherapy [92]. Similar results were also identified in serum but with more pronounced negative impact on survival of metastatic CRC patients [93].

Hypermethylation of these two genes *IKZF1* and *BCAT1* in plasma of CRC patients after surgery increased their risk of residual disease and subsequently the risk of recurrence [94]. Several studies have explored the use of these 2 methylated ctDNA in plasma of CRC patients who are in remission or after treatment in order to detect recurrence [95–97]. The sensitivity and the odds ratio of these two methylated ctDNA test for recurrence were significantly higher than the sensitivity of CEA. Upon adjusting for other predictors of the presence of recurrence, a positive methylated ctDNA of *BCAT1* and *IKZF1* was an independent predictor (odds ratio, 155.7; 95% CI, 17.9–1360.6; *p* < 0.001) in plasma of CRC patients either during surveillance or within 12 months of the confirmation of recurrence [96].

Other prognostic and predictive methylated ctDNA biomarkers that were mentioned only in a single study were methylated ctDNA of somatostatin (*SST*), tachykinin-1 (*TAC1*) and adenomatous polyposis coli (*APC*). Two genes that are usually downregulated in CRC were studied as circulating methylated biomarker: *SST* gene encodes a well-characterized gastrointestinal neuroendocrine and growth regulatory peptide that acts as a tumor suppressor gene and its promoter is silenced in CRC [98]. High methylation of *SST* ctDNA in sera of CRC patients who only underwent elective curative surgical resection is associated with low cancer-specific survival especially in stage III and with low DFS as well as higher risk of recurrence [99]. Patients having hypermethylation levels in their serum of *APC* gene (encodes tumor suppressor that destabilizes and degrades β‑catenin) preoperatively and *TAC1* gene (encodes a neuroendocrine gastrointestinal peptide) at 6-month follow-up had unfavorable OS particularly in early stage CRC and poor DFS respectively [81, 93].

Panels of methylated ctDNA were examined to predict the prognosis of CRC patients. A recent study of a panel of 13 methylated DNA (*FER1L4*, *VAV3, CHST2, DTX1, PDGFD, SMBT2, QKI, ZNF568, ANKRD13B, ZNF671, CNNM1, GRN2D, and JAM3*) in plasma from 40 cases and 60 healthy controls detected recurrent/metastatic colorectal cancer especially in patients with liver or lung metastasis, with 90% sensitivity, 90% specificity, and an AUC of 0.96 [59]. A five gene prognostic methylation panel consisting of *MYO1G, CALML4, GCET2, KLF3, and ATXN1* genes were identified using targeted bisulfite sequencing of 801 CRC patients and 1021 healthy controls after marker selection based on comparison of CRC tissue DNA methylation data from TCGA and normal blood leukocyte methylation data from an aging study [57]. In spite of the inconsistencies in sample types of CRC and controls that might increase data deviation in marker screening, Luo et al*.* built a prognostic prediction model using the five genes and formulated combined prognosis score (cp-score) that takes into consideration the training and validation datasets. High cp-score in plasma of CRC patients was associated with poor prognosis and was an independent prognostic risk factor in a multivariable analysis in both training and validation cohorts. This cp-score was even superior to other prognostic risk factors (CEA status, TNM stage, and primary tumor location) [57]. Another five gene methylation panel of *EYA4, GRIA4, ITGA4, MAP3K14-AS1, MSC* genes was first discovered in genome-wide methylation microarrays of CRC cell lines and validated in tumor tissue and ctDNA from metastatic CRC patients [100]. Hypermethylation of this panel in the pretherapy plasma of metastatic CRC patients receiving regorafenib treatment was associated with worse OS and increased risk of progression, while its level during regorafenib treatment or its dynamic change was correlated with shorter PFS [101]. Hence its longitudinal assessment as a dynamic biomarker could be utilized relatively early during the treatment of metastatic CRC patients, before radiological assessment, to identify those with a negative prognosis.

## Challenges and recommendations

**Fig. 1:**
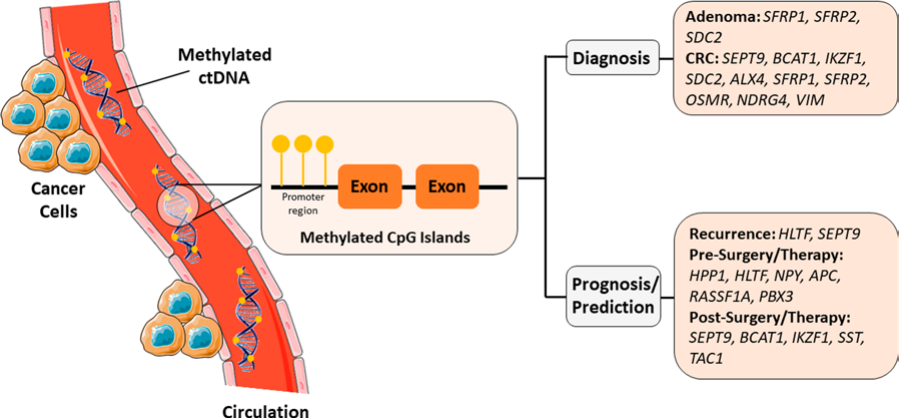
Summary of potential methylated ctDNA in colorectal cancer patients as diagnostic, prognostic, and predictive circulating biomarkers. This figure was designed using some images from Servier Medical Art by Servier, licensed under a Creative Commons Attribution 3.0 Unported License (https://smart.servier.com/)

**Fig. 2:**
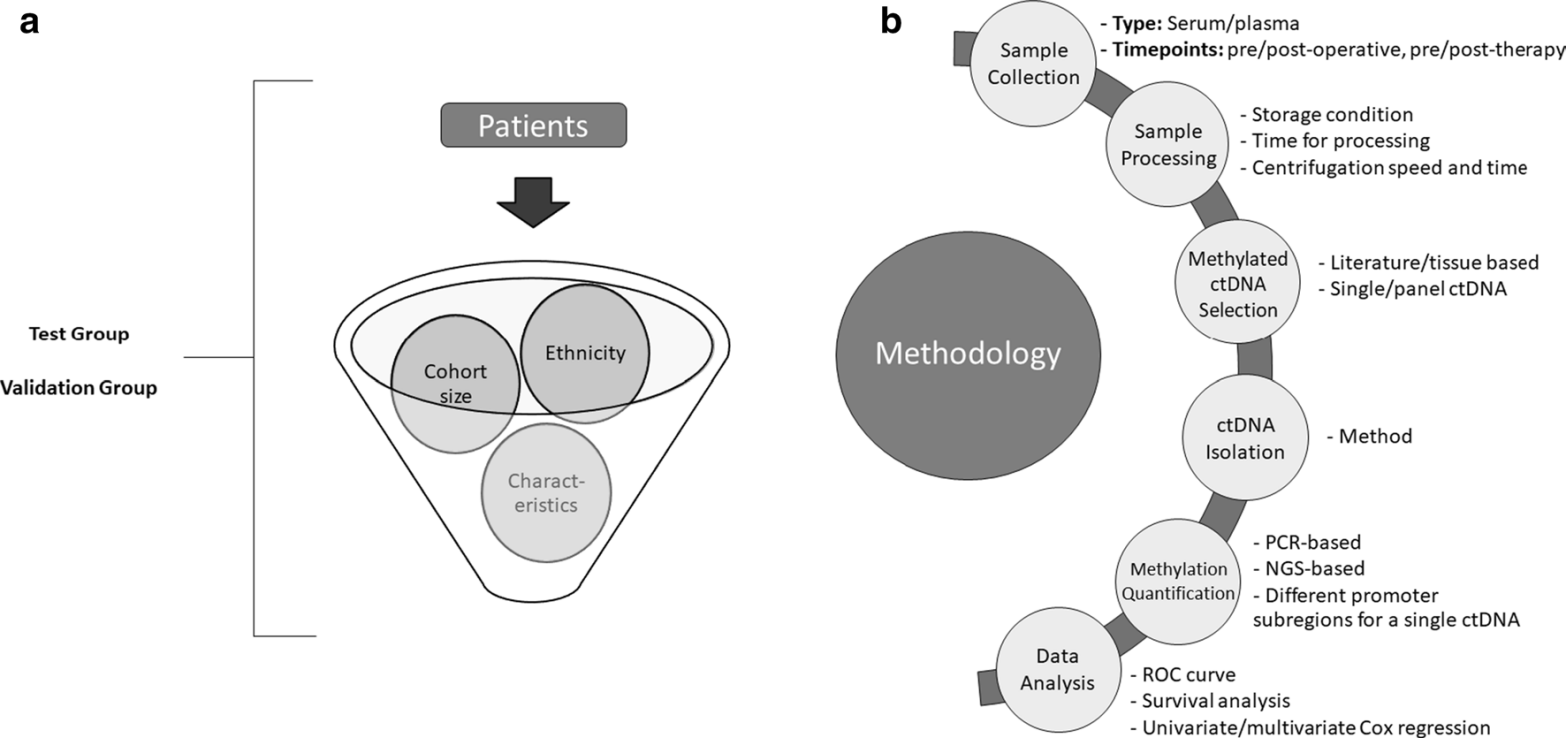
Guidelines for identifying methylated ctDNA optimal biomarker for CRC

Detection of circulating tumor-derived methylated DNA biomarkers in CRC might help in diagnosis, prognosis, and prediction (Fig. 1). Since methylated ctDNA is a stable molecule with high clinical sensitivity and ease in detection in a minimally invasive manner, several studies have investigated the performance of a single or a panel of such potential blood-based markers, but further essential standardization and fine-tuning are required. Some studies followed checklists to ensure complete and transparent reporting such as the REMARK (REporting recommendations for tumor MARKer prognostic studies) consisting of 20 items to report for published tumor marker prognostic studies [88], STROBE-ME (STrengthening the reporting of OBservational studies in Epidemiology—Molecular Epidemiology) including 22 items to be reported in epidemiological studies [102], STARD (STAndards for the Reporting of Diagnostic accuracy studies) initiative that lists 30 items for diagnostic accuracy studies [103, 104] and TRIPOD (Transparent reporting of a multivariable prediction model for individual prognosis or diagnosis) which is 22 checklist items deemed essential for transparent reporting of a prediction model study [105]. However, none of them go into thorough technical molecular details that are essential for identifying biomarkers (Fig. 2).

To begin with, the methodology for methylated ctDNA processing, isolation and quantification should be fully adjusted. A recent systematic review that discusses the methodological factors influencing recovery and quantification of circulating free DNA has developed recommendations for optimal conditions regarding blood specimen type, storage conditions of blood and time to processing, centrifugation speed and time, and method of ctDNA isolation and quantification [106]. The choice of plasma or serum can affect the levels of ctDNA. Higher levels of ctDNA seem to be present in serum compared to plasma due to contamination from genomic DNA which are large DNA shedding from leukocyte lysis during the clotting process. A study in 2020 using digital droplet PCR revealed that ctDNA was less frequently detected in serum of cancer patients than that in plasma where tumor-derived DNA is less diluted [107]. Processing of blood sample should be within 6 h and double centrifugation are needed [108]. This step is crucial to be done as quick as possible especially that methylated ctDNA is highly influenced by the proportion of cell types present in the blood sample [109]. Choosing the best method to detect DNA methylation must take into consideration: amount of DNA needed, robustness and simplicity of the method and cost. Several methods were reported in the different sections of this review. A recent review has compared four commonly used methylation methods: methylation-specific restriction endonucleases (MSRE) analysis, pyrosequencing, methylation-specific high-resolution DNA melting (MS-HRM) and quantitative methylation-specific polymerase chain reaction (qMSP) [110]. They concluded that pyrosequencing and MS-HRM are the most convenient methods. Pyrosequencing analyzes every methylated region in a chosen location, but it is a bit costly. MSHRM is a quick, cheap, very accurate and easy PCR-based method. On the other hand, MSRE is an expensive assay not suitable for intermediately methylated regions. Its analysis is based on a methylation-specific digestion of DNA that does not need DNA bisulfite conversion as the other methods. qMSP is the least accurate and time-consuming method especially that its primers are designed specifically for methylated and unmethylated alleles of a chosen region. Other enhanced methods were developed like MethyLight PCR that amplifies bisulfite-converted DNA in combination with fluorescently labeled probes that hybridize specifically to a predefined DNA methylation pattern. Combination of multiple biomarkers has been used to improve sensitivity of diagnostic tests such as ColoDefense assay which is multiplex qPCR that was used to detect the methylation of *SEPT9* and *SDC2,* from purified bisulfite-converted DNA, simultaneously [16, 17]. Recently, digital droplet PCR has been reported to be more sensitive than real time PCR for detection of low abundant targets such as methylated ctDNA with higher precision, greater accuracy, and technical simplicity [111]. It might be a better technology to detect AA since detection of abnormal methylation in blood depends on the methylation level in abnormal tissue and the amount of DNA that can be released into circulation from cell turnover [82, 110]. Another novel way to improve the detection rate of methylated ctDNA is to use multiple DNA methylation markers of different subregions in the promoter rather than one subregion as done by Jin et al*.* for *SEPT9*. As such, testing multiple DNA methylation markers will overcome any variations in DNA methylation patterns and make it more sensitive particularly in patients with early-stage cancer or early in recurrence [29]. Notably, this method of multiple markers was even more sensitive in postoperative and follow-up plasma samples than targeted NGS covering 532 cancer-related genes for plasma ctDNA detection.

In addition to that, most of the studies identifying blood-based methylated DNA biomarkers were examined on a small size of patients of different characteristics but on specific ethnic groups. A study has shown that the global leukocyte DNA methylation can differ by gender and race/ethnicity in peripheral blood which should be taken into consideration when choosing a biomarker [112]. For instance, most of the studies on *HLTF* and *HPP1* methylated ctDNA were done in the same ethnic groups in Germany which should be further validated in other ethnic groups. Studies have shown that DNA methylation detected in CRC tissues could be divergent between different populations [113, 114]. For example, a study conducted on 51 Iranian and 51 African-American CRC tissues showed that the latter had higher *GPNMB*, *ICAM5*, and *CHD5* promoter methylation levels than Iranians [115]. Even though these studies were done on tissues, some studies showed concordance between circulating ctDNA and tumor tissue methylation profile of specific genes. When comparing the methylation status between tissues and their matched plasma/serum, some markers showed high concordance like *RUNX3* (94.4%) and *SFRP1* (94.3%) [116] while others showed low concordance like *OSMR* (48%) [42]. Moreover, *SEPT9* showed a positive correlation between tumor tissues and their matched plasma with a *p*-value of 0.001 [21] and *NEUROG1* showed a positive correlation between tumor tissues and their matched serum in 18 out of 35 samples [49], suggesting that some methylated ctDNA might be accessible biomarkers for CRC detection in circulation. In addition, DNA methylation pattern might be affected by the age of the studied population. As mentioned previously, a model of seven methylated gene promoter regions (*ALX4*, *BMP3*, *NPTX2*, *RARB*, *SDC2*, *SEPT9*, and *VIM*) and the covariates, female gender, and age greater than 66 years, had the ability to distinguish colorectal patients from healthy individuals [13]. Another study showed that CRC subjects older than 60 years had significantly higher methylation levels of *SEPT9* in the plasma as compared to the younger subjects (40.1% vs 24.2%) [25]. On the other hand, the presence of methylated *NDRG4*, *GATA5*, *FOXE1*, and *SYNE1* in plasma was not associated with age and gender [50]. Hence, further validation studies must be performed on a larger sample size of different ethnicities, different age groups, and specific characteristics (receiving specific treatment or with specific mutations or of particular stages). Some of studies were restricted by a relatively short clinical follow-up, so further investigations with longer clinical monitoring are still required to assess the reliability of prognostic biomarkers in clinical decision-making for patients. In the studies investigating the prognosis of methylated ctDNA, defined blood sampling intervals are required to clarify the best time to determine the ctDNA status in terms of predicting. For example, direct postoperative level of methylated ctDNA may be affected by severe inflammation or stress after surgery which may lead to increased cell turnover rate and subsequently temporary increase of methylated ctDNA level [82]. Thus, longitudinal monitoring post-resection must be done for each biomarker. Since CRC recurrence and tumorigenesis, may develop through various pathways, different methylation markers become detectable at different time frames, so a combination panel of methylation markers rather than single one for monitoring is required [81].

Interestingly, for prognostic and predictive methylated ctDNA, each study investigating utilized a different method other than multivariate analysis to increase the robustness of these markers. Akaike information criterion (AIC) was used to compare different Cox models by evaluating the performance of models either combining any of the parameters or testing parameters alone [76, 79]. Small AIC indicates better models. For instance, a study compared a model including established clinical parameters alone, like the mutational status, grading, Eastern Cooperative Oncology Group performance status, and tumor load or in combination with the m*HPP1* ctDNA and/or CEA levels in blood sample before and after therapy [79]. Another powerful statistical tool called the propensity score (PS) method was used to decrease the likelihood of confounding bias when analyzing observational data from a cohort study in order to obtain results closer to a completely randomized control study [117]. This score is more practical and statistically more efficient than other conventional strategies such as matching on covariates, stratified analyses, or multivariate statistical methods [118]. Another method that discriminated accurately between patients of different prognosis was combined prognostic score that multiplies the unbiased coefficient estimates (from the trained model) and the marker methylation value matrix in both the training and validation datasets [57].

## Conclusion

Based on this review, methylated ctDNAs have a promising future as a circulating biomarker for CRC diagnosis, prognosis, and prediction. Therefore, these biomarkers could help us improve CRC early detection and patient care and surveillance after large-scale clinical trials and validations. This minimally invasive liquid biopsy biomarker still requires not only optimization and standardization of blood collection, ctDNA isolation, and quantification but also the evaluation of its performance as a biomarker to encourage its use in clinical practice. The performance of the biomarkers is affected by the cutoffs considered to determine the sensitivity and specificity, the statistical analyses between the biomarker and the needed outcome, the low sample size as well as the ethnic and age group of the participants. Furthermore, the development of algorithms or scores, which increase the robustness of these markers through taking into consideration the confounding factors, will be a further tool to improve the current efficacy of this biomarker. Finally, more research is needed to find the predictive role of circulating DNA methylation since little is reported on this rising potential.

## Data Availability

Not applicable.

## Abbreviations

AA: Advanced adenoma
AIC: Akaike information criterion
*ALX4*: Aristaless-like homeobox 4
*APC*: Adenomatous polyposis coli
AUC: Area under the curve
*BCAT1*: Branched-chain amino acid transaminase 1
*BMP3*: Bone morphogenetic protein 3
CA19-9: Carbohydrate antigen
cd-score: Combined diagnostic score
CEA: Carcinoembryonic antigen
cp-score: Combined prognosis score
CRC: Colorectal cancer
CT: Computed tomography
CTC: Circulating tumor cells
ctDNA: Circulating tumor DNA
EMT: Epithelial-to-mesenchymal transition
FDA: Food and Drug Administration
FIT: Fecal immunohistochemical test
FOBT: Fecal occult blood test
*GATM*: Glycine amidinotransferase, mitochondrial
GEO: Gene Expression Omnibus
*HLTF*: Helicase-like transcription factor
*HPP1*: Hyperplastic polyposis 1
*IKZF1*: IKAROS family zinc finger 1
*IRF4*: Interferon regulatory factor 4
LDH: Lactate dehydrogenase
MAPK: Mitogen-activated protein kinase
MCTA-Seq: Methylated CpG tandem amplification and sequencing
m*SEPT9*: Methylated *Septin 9*
MS-HRM: Methylation-specific high-resolution DNA melting
MSRE: Methylation-specific restriction endonucleases
NAA: Non-advanced adenoma
NCI: National Cancer Institute
*NPTX2*: Neuronal pentraxin 2
OS: Overall survival
*OSMR*: Oncostatin M receptor
PCR: Polymerase chain reaction
PDR: Positive detection rate
PFS: Progression-free survival
PS: Propensity score
qMSP: Quantitative methylation-specific real-time PCR
*RARB*: Retinoic acid receptor beta
REMARK: REporting recommendations for tumor MARKer prognostic studies
ROC: Receiver operating characteristic
*SDC2*: Syndecan-2
SEER: Surveillance, Epidemiology, and End Results
*SFRP1*: Secreted frizzled-related protein 1
*SFRP2*: Secreted frizzled-related protein 2
SSP: Sessile serrated adenomas/polyps
*SST*: Somatostatin
STARD: STAndards for the Reporting of Diagnostic accuracy studies
STROBE-ME: STrengthening the reporting of OBservational studies in Epidemiology—Molecular Epidemiology
*TAC1*: Tachykinin-1
TCGA: The Cancer Genome Atlas
*TJP2*: Tight junction protein 2
TRIPOD: Transparent reporting of a multivariable prediction model for individual prognosis or diagnosis
*VIM*: Vimentin
